# Association of red cell distribution width-to-albumin ratio with ICU outcomes in patients with aortic dissection

**DOI:** 10.3389/fcvm.2026.1909912

**Published:** 2026-10-14

**Authors:** Chen Li, Xingping Lv, Yezhou Shen, Xiaobin Liu, Guoliang Fan, Feng Zhu

**Affiliations:** Department of Critical Care Medicine, Shanghai East Hospital, School of Medicine, Tongji University, Shanghai, China

**Keywords:** albumin, aortic dissection, outcomes, red cell distribution width, red cell distribution width-to-albumin ratio

## Abstract

**Objective:**

Critically ill patients with aortic dissection remain at high risk of adverse ICU outcomes, including ICU mortality. The red cell distribution width-to-albumin ratio (RAR) has been investigated as a prognostic marker in various clinical settings. This study aimed to evaluate the association of RAR with ICU outcomes in patients with aortic dissection.

**Methods:**

Data were extracted from the Medical Information Mart for Intensive Care IV (MIMIC-IV) database. ICU mortality was defined as the primary outcome. Secondary outcomes included sepsis during hospitalization/ICU stay, acute kidney injury (AKI), and renal replacement therapy (RRT) use. Receiver operating characteristic (ROC) curve analysis was used to assess the discriminative performance of RAR for ICU mortality. The optimal RAR cutoff was determined using Youden's index and was considered exploratory. Calibration plots and decision curve analysis were used to evaluate model performance.

**Results:**

RAR showed modest discrimination for ICU mortality (AUC: 0.66; 95% CI: 0.552–0.765). The exploratory cutoff value of RAR was 5.182. Compared with the low-RAR group, the high-RAR group had higher ICU mortality (21.8% vs. 6.7%; *P* < 0.001), a higher prevalence of sepsis during hospitalization/ICU stay (82.8% vs. 61.1%; *P* < 0.001), and more frequent RRT use (33.3% vs. 9.4%; *P* < 0.001). The AUC increased from 0.706 for SOFA alone to 0.731 for SOFA plus RAR, indicating only limited incremental discrimination.

**Conclusion:**

Higher early-ICU RAR was associated with ICU mortality and other adverse ICU outcomes in patients with aortic dissection, but its discrimination was modest, its incremental value beyond SOFA was limited, and external validation is required.

## 1 Introduction

Aortic dissection is a rare but life-threatening cardiovascular emergency associated with substantial morbidity and mortality (1). Patients admitted to the intensive care unit (ICU) with aortic dissection often present with marked physiological derangement and remain at high risk of adverse clinical outcomes, including organ dysfunction and death (2–4). Early identification of higher-risk patients may assist clinical assessment and risk stratification in this population.

Red blood cell distribution width (RDW) reflects erythrocyte size variability and is increasingly recognized as a marker of inflammation and oxidative stress (5, 6). Serum albumin, a negative acute-phase protein, indicates nutritional status and is closely linked to inflammation and oxidative stress modulation (7–10). Red blood cell distribution width-to-albumin ratio (RAR) has recently emerged as a composite biomarker that integrates two routinely available parameters associated with inflammation, nutritional status, and disease severity. Accumulating evidence suggests that elevated RAR is associated with adverse outcomes in a variety of clinical settings, including sepsis, chronic kidney disease, burn injury, and other critically ill populations (11–15). More recently, its clinical relevance has also been explored in inflammatory cardiovascular disorders. For example, elevated RAR has been associated with several clinical parameters in patients with myocarditis and pericarditis, supporting its potential value in cardiovascular inflammatory conditions (16, 17). In addition, recent data from intensive-care cohorts indicate that RAR may be associated with short-term mortality in critically ill patients, further supporting its broader prognostic significance in the ICU setting (18). Collectively, these findings suggest that RAR may reflect a general pathophysiological state characterized by systemic inflammation, nutritional impairment, and overall illness severity, rather than a marker specific to a single disease entity. However, the prognostic value of RAR in critically ill patients with acute aortic dissection remains insufficiently studied. Despite its clinical potential, no studies have examined RAR in the prognosis of aortic dissection. This study aims to assess the prognostic value of RAR in aortic dissection patients, providing a novel tool for risk stratification.

## 2 Methods

### 2.1 Study population

This study used the MIMIC-IV database, which was approved by the institutional review boards of Beth Israel Deaconess Medical Center and the Massachusetts Institute of Technology (No.2001P001699). Because the MIMIC-IV database contains de-identified patient data and this study was a secondary analysis of publicly available de-identified data, individual informed consent was not required. Researchers were certified through the CITI program (ID:53376570) and were granted access to the database according to institutional requirements. We selected all patients with aortic dissection (*n* = 496). Individuals lacking RDW and ALB data were excluded (*n* = 229). Ultimately, data from 267 participants were analyzed (Figure 1). Patients with missing RDW or albumin values were excluded from the primary analysis. To evaluate potential selection bias introduced by this exclusion, we compared available baseline characteristics and mortality outcomes between included and excluded patients.

**Figure 1:**
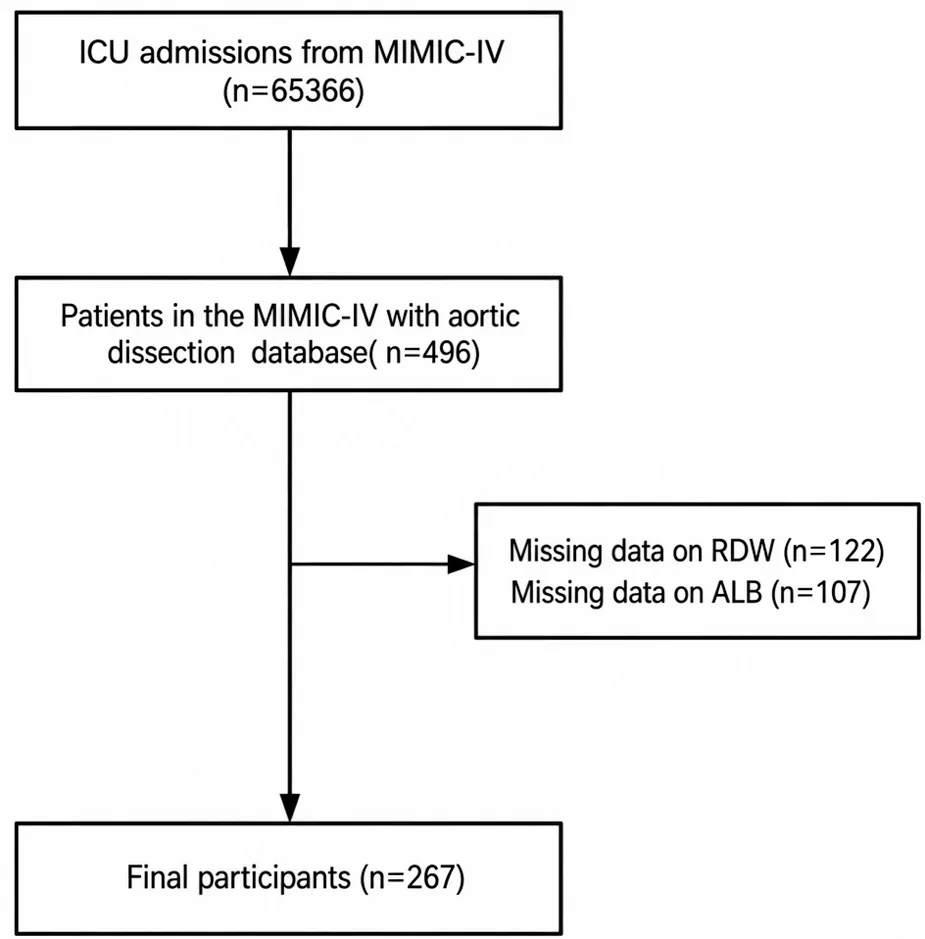
Flow diagram of patient selection.

### 2.2 Data collection

Data were extracted using Navicat Premium (v15.0). Patients diagnosed with aortic dissection were screened, and data on age, sex, ICU stay, and ICU mortality were collected from the MIMIC-IV database. Information on AKI, RRT, and infections was also recorded. Laboratory values (e.g., glucose, WBC, RBC, RDW, platelets, albumin, lactate, creatinine, INR), vital signs (heart rate, MAP, respiratory rate), and clinical scores (SOFA, OASIS, SAPS II, APS III, Charlson) were obtained within 24 h of ICU admission. For repeated measurements, mean values were used. RAR was calculated as RDW (%) divided by albumin (g/dL). The first available RDW and albumin values obtained after ICU admission as the baseline exposure. Only first-time ICU admissions were included.

### 2.3 RAR definition and grouping

The “high-RAR” and “low-RAR” groups were defined according to the optimal cutoff derived from the ROC curve using the Youden index.

### 2.4 Outcome definitions

The primary outcome was ICU mortality, defined as death occurring during the ICU stay. The secondary outcomes were sepsis during hospitalization/ICU stay, acute kidney injury (AKI), and renal replacement therapy (RRT) use during ICU stay. Sepsis was identified using available infection- and organ dysfunction-related data from the MIMIC-IV database and was defined as closely as possible according to the Sepsis-3 framework. AKI was defined mainly according to the creatinine-based Kidney Disease: Improving Global Outcomes (KDIGO) criteria. Baseline creatinine was defined as the most recent measurement before ICU admission when available; otherwise, the first creatinine value after ICU admission was used. RRT was defined as documented receipt of continuous or other renal replacement therapy during ICU stay. Because urine output and some clinical variables were not consistently available, some outcome misclassification may have occurred. Moreover, exact onset times of sepsis, AKI, and RRT were not uniformly available; therefore, these outcomes were analyzed as ICU/hospitalization-related clinical outcomes rather than strictly incident events occurring after baseline RAR measurement.

### 2.5 Statistical analysis

The normality of continuous variables was assessed using the Kolmogorov–Smirnov test. Variables conforming to a normal distribution were presented as mean ± standard deviation and compared using the independent samples *t*-test. Continuous variables not following a normal distribution were described using median and interquartile range (IQR), and group comparisons were performed using the Wilcoxon rank-sum test. Categorical variables were expressed as frequencies and percentages, and analyzed by the chi-square test. To further explore the potential dose-response relationship between RAR and the primary outcome, restricted cubic spline (RCS) analysis based on multivariable logistic regression was performed. Four knots were placed at the 5th, 35th, 65th, and 95th percentiles of the RAR distribution. The spline model was adjusted for sex, age, hypertension, diabetes mellitus, chronic kidney disease, chronic obstructive pulmonary disease, white blood cell count, aspartate aminotransferase, blood urea nitrogen, and serum creatinine. Odds ratios (ORs) with 95% confidence intervals (CIs) were plotted to visualize the shape of the association. *P* values for overall association and nonlinearity were calculated, with *P* < 0.05 considered statistically significant. To evaluate the association between RAR and the primary outcome, univariable and multivariable logistic regression analyses were performed. A stepwise modeling strategy was used to assess the robustness of the association. Model 1 was unadjusted. Model 2 was adjusted for age and sex. Model 3 was further adjusted for major comorbidities, including hypertension, diabetes mellitus, chronic kidney disease, and chronic obstructive pulmonary disease. Model 4 was additionally adjusted for laboratory variables reflecting inflammatory status and organ function, including white blood cell count, aspartate aminotransferase, blood urea nitrogen, and serum creatinine. Odds ratios (ORs) with 95% confidence intervals (CIs) were reported. A two-sided *P* value < 0.05 was considered statistically significant. The cutoff value derived from the ROC curve using the Youden index was considered exploratory, as it was derived and evaluated within the same cohort. Calibration plots were employed to evaluate the calibration performance of the model. Clinical decision curve analysis (DCA) was conducted to assess its clinical applicability. Internal validation was performed using bootstrap resampling with 1,000 samples. In each bootstrap sample, the model was refitted and evaluated in both the bootstrap sample and the original dataset to estimate optimism. Optimism-corrected estimates were calculated for the C-statistic, calibration slope, and Brier score. Based on the exploratory cutoff point determined from the ROC curve, Kaplan–Meier survival curves were constructed to compare time to ICU death between the high- and low-RAR groups. For this analysis, time zero was defined as the time of ICU admission. Patients were followed from ICU admission until ICU death or ICU discharge, whichever occurred first. Patients who were discharged alive from the ICU were treated as censored at the time of ICU discharge. Because live ICU discharge may act as a competing event rather than non-informative censoring, the Kaplan–Meier analysis and Cox proportional hazards model were used for exploratory descriptive purposes only. The hazard ratio (HR) reported from the Cox model was unadjusted.

## 3 Results

### 3.1 Baseline characteristics

A total of 94,458 admission records were identified in the MIMIC-IV database. After a rigorous screening process, 267 cases of aortic dissection were ultimately included. The data selection flowchart is presented in Figure 1. Clinical characteristics stratified by the RAR cutoff value are summarized in Table 1. Among the 267 participants, 180 (67.4%) were assigned to the low RAR group, and 87 (32.6%) to the high RAR group. The high RAR group exhibited significantly lower levels of hemoglobin, albumin, and red blood cell count, with hemoglobin at 8.7 g/dL (IQR, 7.85–10.2) vs. 10.7 g/dL (8.9–12.22; *p* < 0.001), albumin at 2.6 g/dL (2.3–2.8) vs. 3.3 g/dL (3.0–3.6; *p* < 0.001), and red blood cell count at 3.04 × 10^12^/L (2.65–3.52) vs. 3.52 × 10^12^/L (2.96–4.12; *p* < 0.001), respectively. Conversely, the high RAR group showed elevated levels of aspartate aminotransferase (AST), creatinine, blood urea nitrogen (BUN), and international normalized ratio (INR), with AST at 73.0 U/L (33.5–189.0) vs. 39.0 U/L (23.0–79.25; *p* < 0.001), creatinine at 1.2 mg/dL (0.9–1.5) vs. 1.0 mg/dL (0.8–1.3; *p* = 0.012), BUN at 21.0 mg/dL (16.0–26.5) vs. 18.0 mg/dL (14.0–23.0; *p* = 0.013), and INR at 1.5 (1.2–1.8) vs. 1.35 (1.2–1.6; *p* = 0.009). Additionally, the high RAR group had longer hospital length of stays (16.07 days [IQR, 8.52–25.88] vs. 11.94 days [7.15–18.08]; *p* = 0.026), longer ICU stays (7.77 days [3.42–16.47] vs. 4.64 days [2.68–9.80]; *p* = 0.003), and prolonged mechanical ventilation duration (138.0 h [51.91–294.63] vs. 81.84 h [32.17–161.44]; *p* = 0.003). Moreover, the high RAR group exhibited higher SOFA, OASIS, SAPS II, APS III, and Charlson comorbidity scores compared to the low RAR group. (*P* < 0.05, as shown in Table 1).

**Table 1:** Comparison of baseline data and clinical indicators between two groups.

Variables	Total (*n* = 267)	Low RAR (≤5.182) (*n* = 180)	High RAR (>5.182) (*n* = 87)	*P*
Age, M (Q_1_, Q_3_)	70.00 (59.00, 79.00)	70.00 (58.00, 78.00)	70.00 (61.00, 80.50)	0.228
White blood cell count (x10^9^/L), M (Q_1_, Q_3_)	10.70 (7.90, 14.70)	11.00 (8.40, 14.80)	9.80 (7.05, 14.35)	0.080
Red blood cell count (x10^12^/L), M (Q_1_, Q_3_)	3.37 (2.79, 3.95)	3.52 (2.96, 4.12)	3.04 (2.65, 3.52)	**<**.**001**
Platelet (x10^9^/L), M (Q_1_, Q_3_)	146.00 (105.00, 196.00)	150.50 (107.00, 204.00)	136.00 (95.50, 194.50)	0.244
Hemoglobin (g/L), M (Q_1_, Q_3_)	10.00 (8.40, 11.70)	10.70 (8.90, 12.22)	8.70 (7.85, 10.20)	**<**.**001**
Albumin (mg/dL), M (Q_1_, Q_3_)	3.10 (2.70, 3.50)	3.30 (3.00, 3.60)	2.60 (2.30, 2.80)	**<**.**001**
Glucose (mg/dL), M (Q_1_, Q_3_)	128.00 (109.50, 161.00)	127.00 (108.00, 159.00)	132.00 (110.50, 172.50)	0.141
Lactate (mmol/l), M (Q_1_, Q_3_)	2.10 (1.40, 3.70)	2.10 (1.30, 2.96)	2.10 (1.45, 4.80)	0.086
INR, M (Q_1_, Q_3_)	1.40 (1.20, 1.70)	1.35 (1.20, 1.60)	1.50 (1.20, 1.80)	**0**.**009**
ALT, M (Q_1_, Q_3_)	29.00 (16.50, 60.00)	27.50 (16.00, 49.25)	30.00 (17.50, 82.00)	0.159
AST, M (Q_1_, Q_3_)	44.00 (25.00, 118.00)	39.00 (23.00, 79.25)	73.00 (33.50, 189.00)	**<**.**001**
BUN, M (Q_1_, Q_3_)	19.00 (14.00, 24.00)	18.00 (14.00, 23.00)	21.00 (16.00, 26.50)	**0**.**013**
Creatinine, M (Q_1_, Q_3_)	1.10 (0.80, 1.40)	1.00 (0.80, 1.30)	1.20 (0.90, 1.50)	**0**.**012**
Heart rate, M (Q_1_, Q_3_)	78.41 (71.02, 89.44)	77.30 (70.30, 88.48)	81.77 (72.69, 91.87)	0.061
MAP, M (Q_1_, Q_3_)	75.41 (71.22, 78.09)	75.41 (71.28, 77.57)	75.41 (70.66, 80.08)	0.339
RR, M (Q_1_, Q_3_)	18.07 (15.96, 20.11)	18.21 (16.04, 19.97)	17.97 (15.81, 20.89)	0.999
Hospital length of stay (days), M (Q_1_, Q_3_)	12.90 (7.58, 21.61)	11.94 (7.15, 18.08)	16.07 (8.52, 25.88)	**0**.**026**
ICU length of stay (days), M (Q_1_, Q_3_)	5.55 (2.79, 11.12)	4.64 (2.68, 9.80)	7.77 (3.42, 16.47)	**0**.**003**
Mechanical ventilation (hours), M (Q_1_, Q_3_)	91.00 (37.78, 205.88)	81.84 (32.17, 161.44)	138.00 (51.91, 294.63)	**0**.**003**
SOFA, M (Q_1_, Q_3_)	6.00 (3.00, 10.00)	5.00 (3.00, 8.00)	9.00 (5.00, 11.00)	**<**.**001**
APSIII, M (Q_1_, Q_3_)	44.00 (36.00, 55.00)	43.00 (33.00, 51.00)	49.00 (39.50, 60.00)	**<**.**001**
SAPSII, M (Q_1_, Q_3_)	39.00 (32.00, 48.00)	37.00 (31.00, 44.00)	45.00 (35.00, 51.00)	**<**.**001**
OASIS, M (Q_1_, Q_3_)	34.00 (29.00, 39.00)	33.00 (28.00, 38.00)	36.00 (32.00, 42.50)	**<**.**001**
CHARLSON, M (Q_1_, Q_3_)	5.00 (4.00, 7.00)	5.00 (3.00, 6.00)	6.00 (4.00, 7.00)	**0**.**003**
RDW, M (Q_1_, Q_3_)	14.10 (13.40, 15.30)	13.80 (13.20, 14.60)	15.20 (14.10, 16.45)	**<**.**001**
Gender, *n* (%)				0.569
F	107 (40.07)	70 (38.89)	37 (42.53)	
M	160 (59.93)	110 (61.11)	50 (57.47)	
Hypertension, *n* (%)				0.694
0	109 (40.82)	72 (40.00)	37 (42.53)	
1	158 (59.18)	108 (60.00)	50 (57.47)	
Diabetes mellitus, *n* (%)				0.075
0	232 (86.89)	161 (89.44)	71 (81.61)	
1	35 (13.11)	19 (10.56)	16 (18.39)	
HF, *n* (%)				0.651
0	204 (76.40)	139 (77.22)	65 (74.71)	
1	63 (23.60)	41 (22.78)	22 (25.29)	
MI, *n* (%)				0.316
0	255 (95.51)	174 (96.67)	81 (93.10)	
1	12 (4.49)	6 (3.33)	6 (6.90)	
MT, *n* (%)				0.975
0	233 (87.27)	157 (87.22)	76 (87.36)	
1	34 (12.73)	23 (12.78)	11 (12.64)	
CKD, *n* (%)				**0**.**037**
0	213 (79.78)	150 (83.33)	63 (72.41)	
1	54 (20.22)	30 (16.67)	24 (27.59)	
AF, *n* (%)				**<**.**001**
0	133 (49.81)	104 (57.78)	29 (33.33)	
1	134 (50.19)	76 (42.22)	58 (66.67)	
Cirrhosis, *n* (%)				0.388
0	263 (98.50)	176 (97.78)	87 (100.00)	
1	4 (1.50)	4 (2.22)	0 (0.00)	
Hepatitis, *n* (%)				0.546
0	265 (99.25)	179 (99.44)	86 (98.85)	
1	2 (0.75)	1 (0.56)	1 (1.15)	
Pneumonia, *n* (%)				0.502
0	212 (79.40)	145 (80.56)	67 (77.01)	
1	55 (20.60)	35 (19.44)	20 (22.99)	
Stroke, *n* (%)				0.244
0	244 (91.39)	167 (92.78)	77 (88.51)	
1	23 (8.61)	13 (7.22)	10 (11.49)	
COPD, *n* (%)				**0**.**004**
0	241 (90.26)	169 (93.89)	72 (82.76)	
1	26 (9.74)	11 (6.11)	15 (17.24)	

Data are expressed as the median (25th–75th percentiles), or number (percentage). BMI, body mass index; ALT, Alanine Aminotransferase; AST, Aspartate Aminotransferase; BUN, Blood Urea Nitrogen; MAP, mean arterial pressure; RR, respiratory rate; RDW, Red Cell Distribution Width; HF, Heart failure; MI, Myocardial infarction; MT, Malignant tumor; CKD, Chronic kidney disease; AF, Atrial fibrillation; COPD, Chronic obstructive pulmonary disease.

Bold values indicate statistical significance (P < 0.05)

### 3.2 Comparison between included and excluded patients

Comparison between included and excluded patients showed substantial differences in baseline characteristics and outcomes (Supplementary Table S1). Overall, included patients appeared to be more severely ill, with higher severity scores, worse laboratory profiles, greater comorbidity burden, and higher rates of sepsis, AKI, mechanical ventilation, and CRRT use.

### 3.3 Multivariable logistic regression analysis

In the crude logistic regression model, RAR was associated with the primary outcome (OR 1.32, 95% CI 1.03–1.71, *P* = 0.030). This association remained significant after adjustment for age and sex (Model 2: OR 1.32, 95% CI 1.02–1.70, *P* = 0.032), further adjustment for comorbidities including hypertension, diabetes mellitus, chronic kidney disease, and chronic obstructive pulmonary disease (Model 3: OR 1.30, 95% CI 1.01–1.69, *P* = 0.049), and additional adjustment for laboratory variables including white blood cell count, aspartate aminotransferase, blood urea nitrogen, and serum creatinine (Model 4: OR 1.32, 95% CI 1.01–1.72, *P* = 0.043). These findings suggest that higher RAR was consistently associated with an increased risk of the outcome across different adjustment models (Table 2).

**Table 2:** Univariable and multivariable logistic regression analyses of the association between RAR and the primary outcome.

Variables	Model1		Model2		Model3		Model4	
	OR (95%CI)	*P*	OR (95%CI)	*P*	OR (95%CI)	*P*	OR (95%CI)	*P*
RAR	1.32 (1.03–1.71)	**0.030**	1.32 (1.02–1.70)	**0.032**	1.30 (1.01–1.69)	**0.049**	1.32 (1.01–1.72)	**0.043**

OR, Odds Ratio; CI, Confidence Interval.

Model1: Crude.

Model2: Adjust: gender, age.

Model3: Adjust: gender, age, Hypertension, Diabetes mellitus, CKD, COPD.

Model4: Adjust: gender, age, Hypertension, Diabetes mellitus, CKD, COPD, White blood cell count, AST, BUN, Creatinine.

AST, Aspartate Aminotransferase; BUN, Blood Urea Nitrogen; CKD, Chronic kidney disease; COPD, Chronic obstructive pulmonary disease.

Bold values indicate statistical significance (P < 0.05)

### 3.4 RAR for ICU mortality

The discriminatory ability of RAR for ICU mortality was modest, with an AUC of 0.66 (95% CI, 0.55–0.76). For comparison, the AUCs of RDW alone and albumin alone were 0.54 (95% CI, 0.44–0.65) and 0.65 (95% CI, 0.54–0.76), respectively, indicating that RAR numerically outperformed RDW but showed similar performance to albumin alone. The Youden index identified an exploratory cutoff value of 5.182 for RAR, corresponding to a sensitivity of 71.19% and a specificity of 61.29% in this derivation cohort (Figure 2). The predictive value of SOFA and RAR + SOFA for ICU mortality was assessed using AUC, yielding an AUC of 0.706 (95% CI, 0.60–0.81) and an AUC of 0.73 (95% CI, 0.63–0.83) (Figure 3). The paired DeLong test showed that this difference was not statistically significant (*P* = 0.148), indicating that adding RAR to SOFA did not provide a statistically significant improvement in discrimination in this cohort.

**Figure 2:**
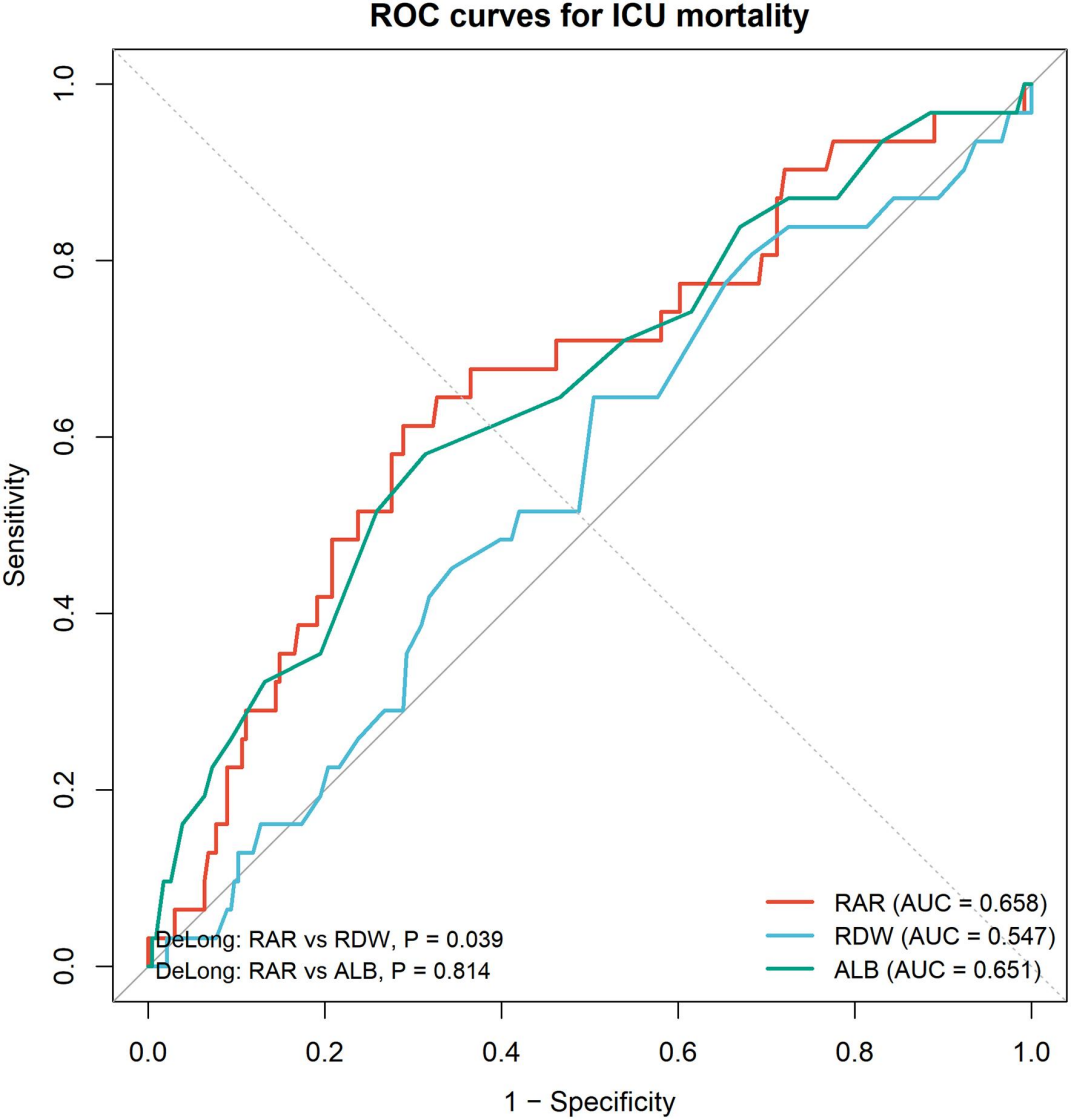
The ROC curve of RAR, RDW and ALB.

**Figure 3:**
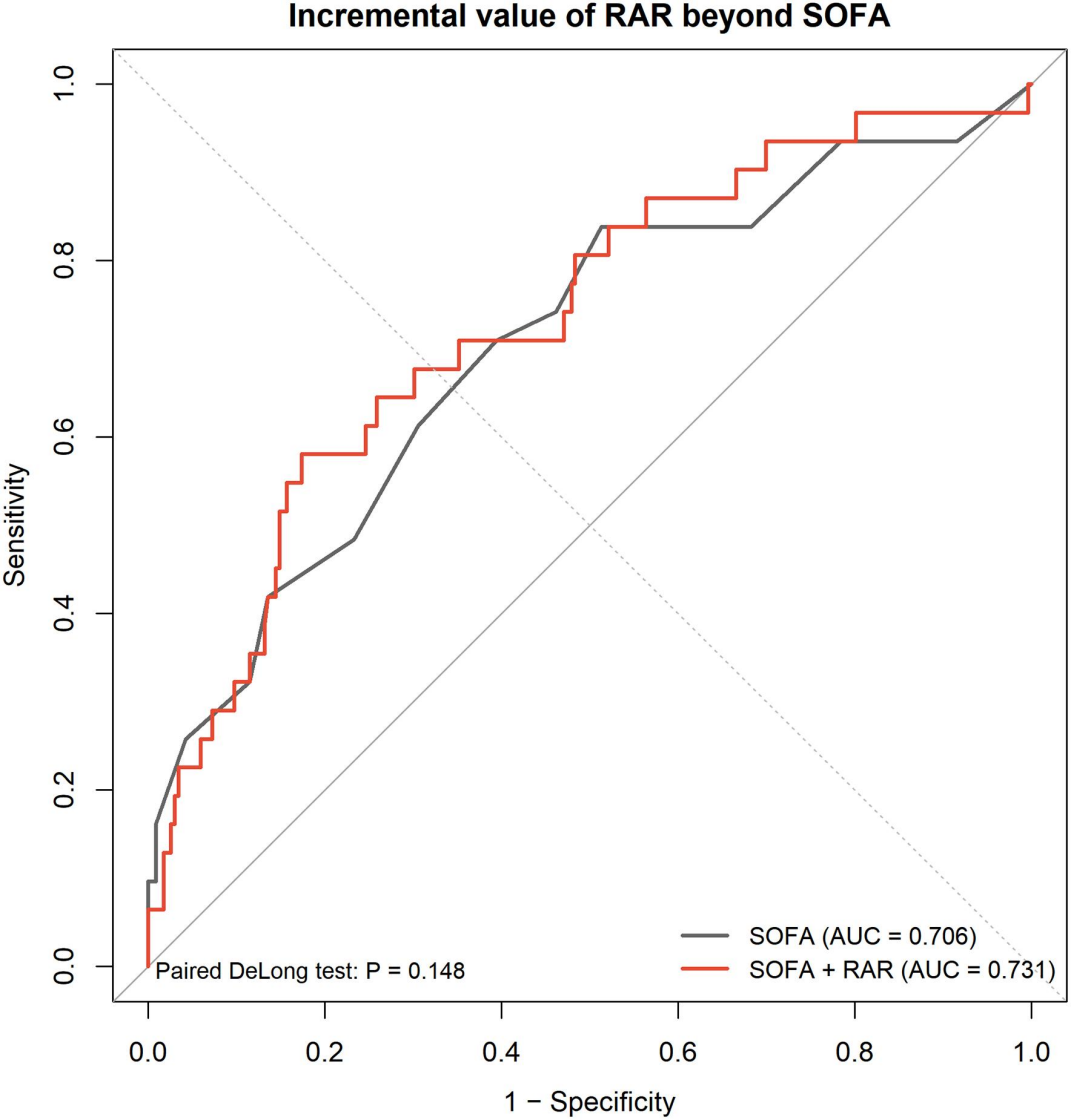
The ROC curve of SOFA and RAR+SOFA.

### 3.5 Calibration and decision curve analysis of the model

The apparent calibration plot of the SOFA + RAR model for ICU mortality showed good agreement between predicted and observed probabilities, with a calibration intercept of 0.000, a calibration slope of 1.000, and a Brier score of 0.0931 (Figure 4A). In bootstrap internal validation using 1,000 resamples, the optimism-corrected C-statistic was 0.72 (95% CI, 0.63–0.82), the optimism-corrected calibration slope was 0.97 (95% CI, 0.59–1.59), and the optimism-corrected Brier score was 0.096 (95% CI, 0.09–0.11). The bootstrap-corrected calibration curve remained generally close to the ideal reference line, although some deviation was observed at lower predicted probabilities (Figure 4B). Decision curve analysis showed that the SOFA + RAR model provided positive net benefit over the treat-all and treat-none strategies across a range of threshold probabilities (Figure 5).

**Figure 4:**
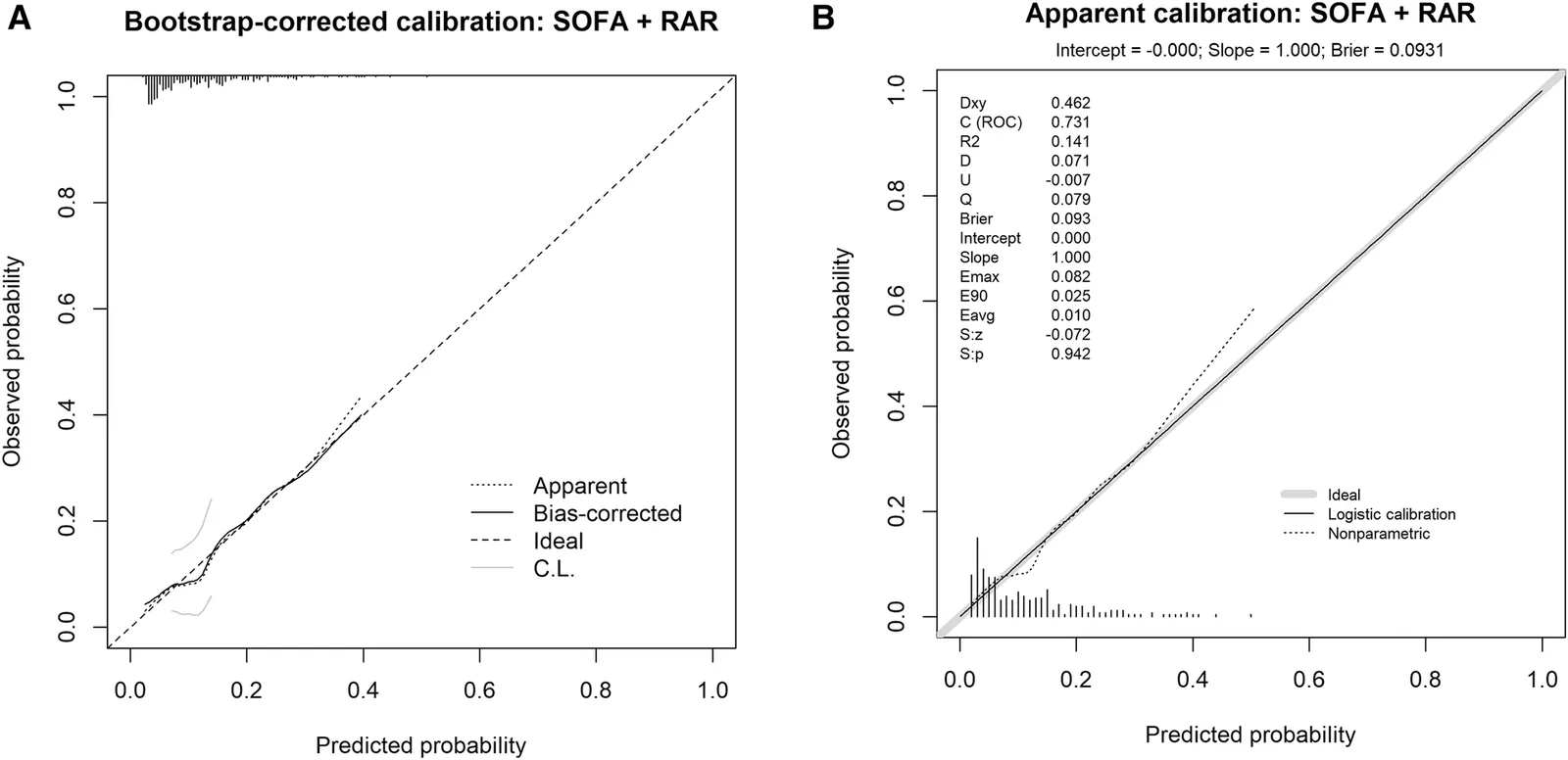
**(A)** Calibration plot of the prediction model; **(B)** Bootstrap-corrected calibration plot.

**Figure 5:**
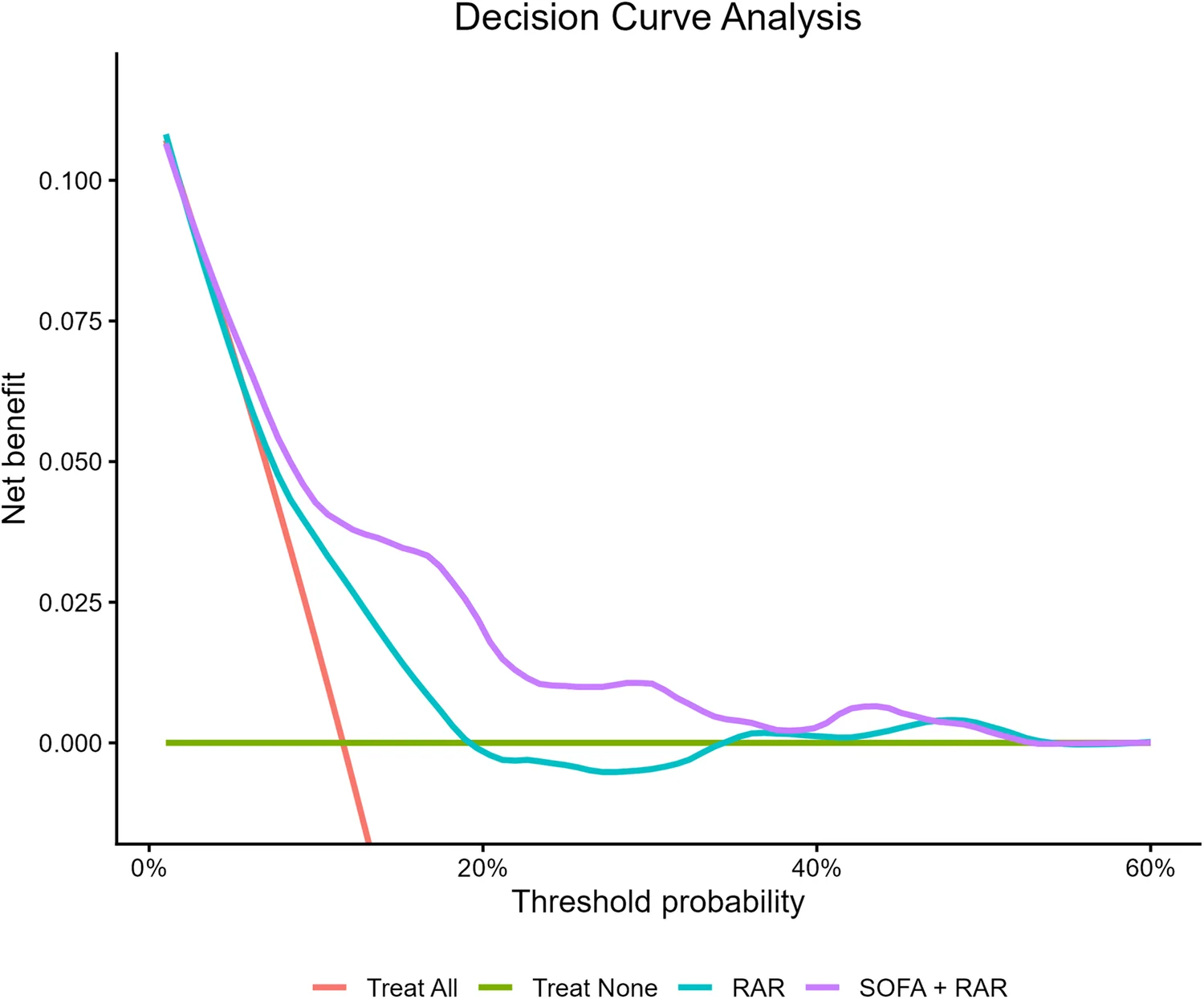
Decision curve analysis of the prediction model.

### 3.6 Association of RAR with ICU clinical outcomes

The high RAR group exhibited a higher ICU mortality rate compared to the low RAR group (21.8% vs. 6.7%; *p* < 0.001). Additionally, the prevalence of sepsis during ICU stay was significantly greater in the high RAR group (82.8% vs. 61.1%; *p* < 0.001), as was the utilization rate of continuous renal replacement therapy (33.3% vs. 9.4%; *p* < 0.001). (*P* < 0.05, as shown in Table 3). Restricted cubic spline analysis based on the fully adjusted logistic regression model showed a significant overall association between RAR and sepsis (*P* for overall < 0.001), with no significant evidence of nonlinearity (*P* for nonlinearity = 0.193), indicating an approximately linear association (Figure 6). A similar approximately linear association was observed between RAR and use of renal replacement therapy (RRT) during ICU stay (*P* for overall < 0.001; *P* for nonlinearity = 0.064) (Figure 7). In contrast, the association between RAR and AKI was significant but nonlinear (*P* for overall = 0.013; *P* for nonlinearity = 0.043) (Figure 8).

**Table 3:** Comparison of outcome and complication between two groups.

Variables	Total (*n* = 267)	Low RAR (≤5.182) (*n* = 180)	High RAR (>5.182) (*n* = 87)	*P*
ICU mortality, *n* (%)				**<**.**001**
0	236 (88.39)	168 (93.33)	68 (78.16)	
1	31 (11.61)	12 (6.67)	19 (21.84)	
Ventilation, *n* (%)				0.579
0	22 (8.24)	16 (8.89)	6 (6.90)	
1	245 (91.76)	164 (91.11)	81 (93.10)	
CRRT, *n* (%)				**<**.**001**
0	221 (82.77)	163 (90.56)	58 (66.67)	
1	46 (17.23)	17 (9.44)	29 (33.33)	
Sepsis, *n* (%)				**<**.**001**
0	85 (31.84)	70 (38.89)	15 (17.24)	
1	182 (68.16)	110 (61.11)	72 (82.76)	
AKI, *n* (%)				0.410
0	17 (6.37)	13 (7.22)	4 (4.60)	
1	250 (93.63)	167 (92.78)	83 (95.40)	

Data are expressed as the median (25th–75th percentiles), median M (Q25, Q75), or number (percentage). CRRT, continuous renal replacement therapy; AKI, acute kidney injury.

Bold values indicate statistical significance (P < 0.05)

**Figure 6:**
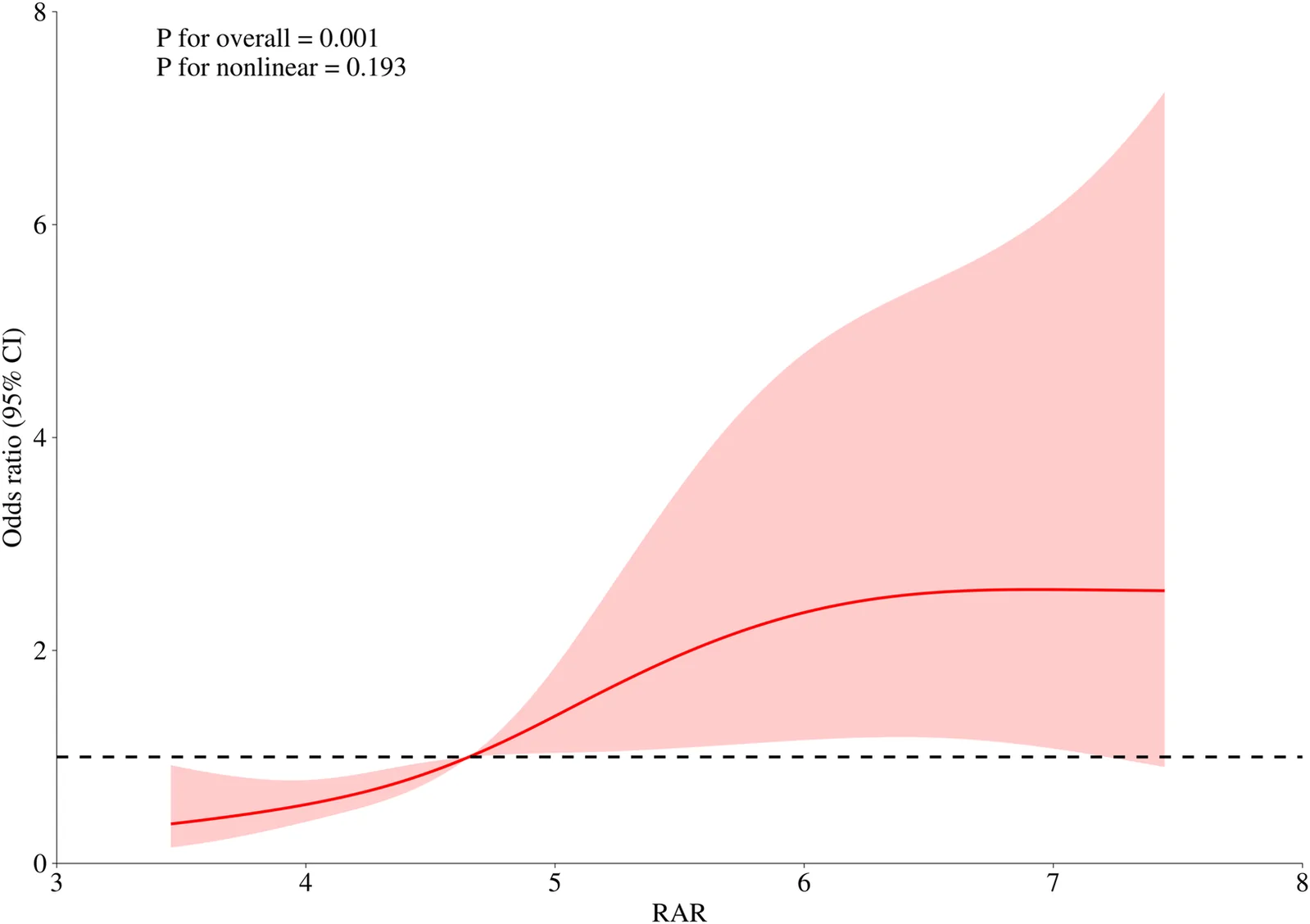
The relationship between RAR and the risk of developing sepsis in patients.

**Figure 7:**
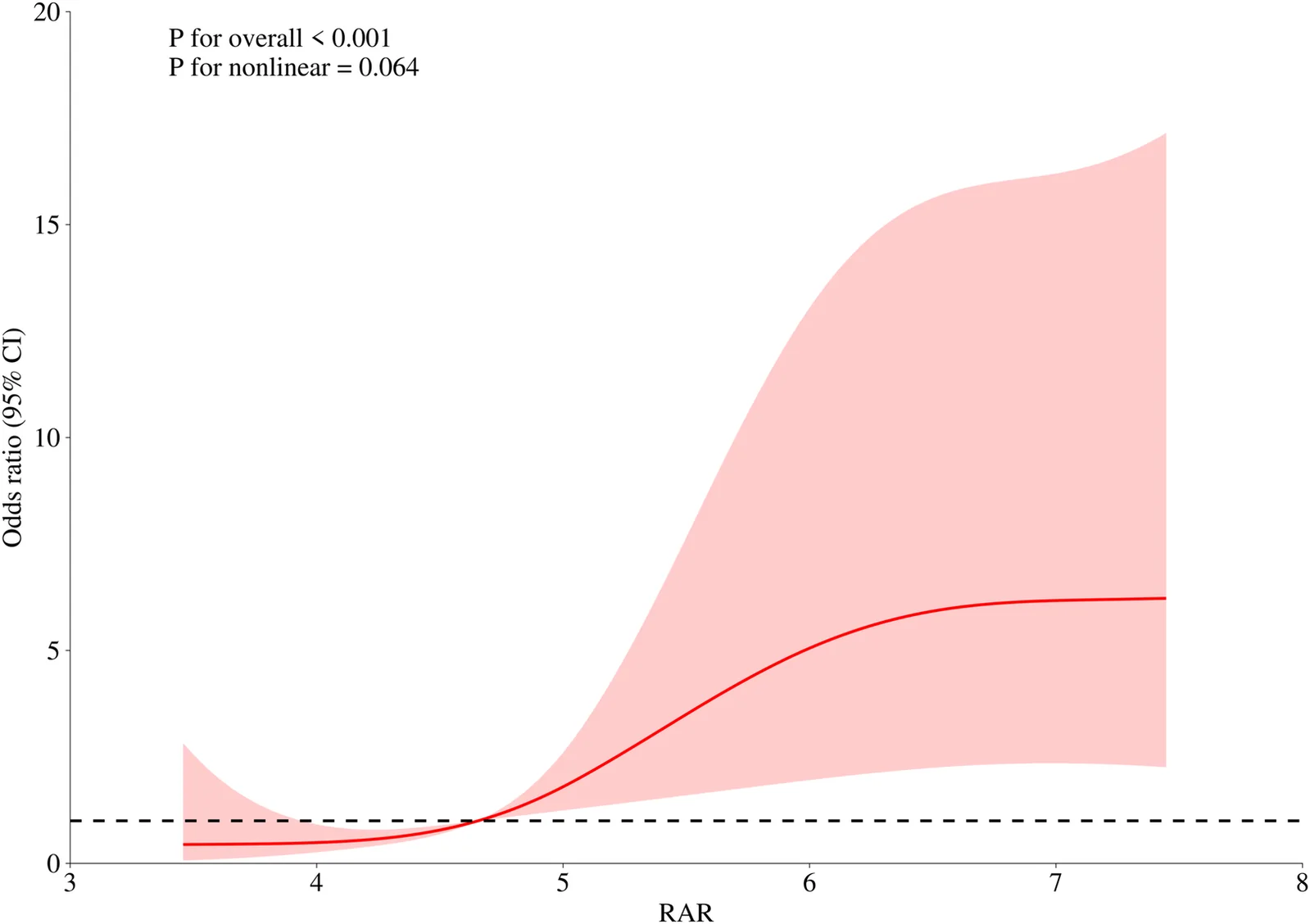
The relationship between RAR and the risk of developing RRT in patients.

**Figure 8:**
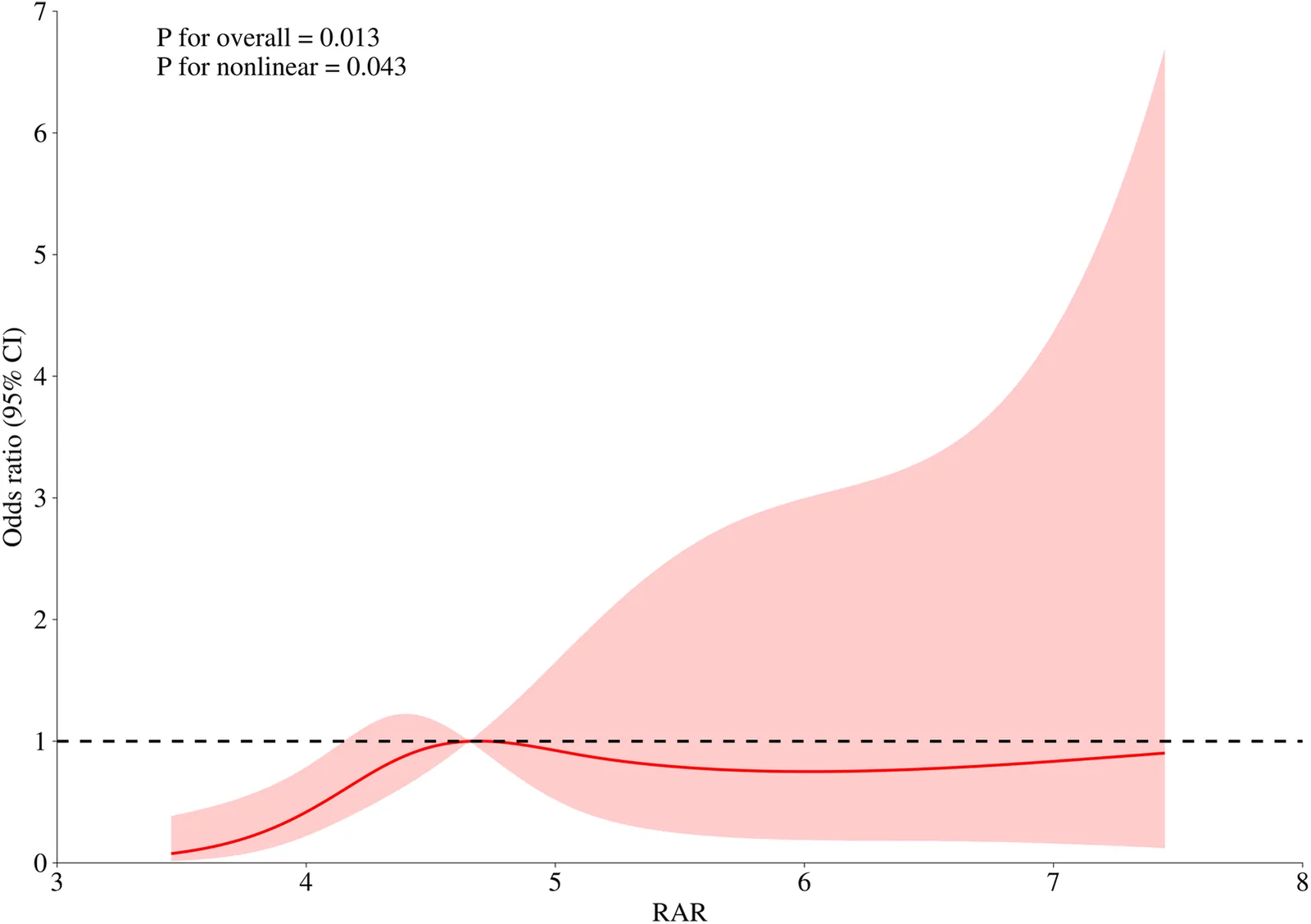
The relationship between RAR and the risk of developing AKI in patients.

### 3.7 Relationship between RAR and scoring systems

The figure demonstrates a positive correlation between RAR levels and APS III, SAPSII, OASIS, SOFA, and Charlson scores, suggesting that RAR may be associated with ICU mortality (Figure 9).

**Figure 9:**
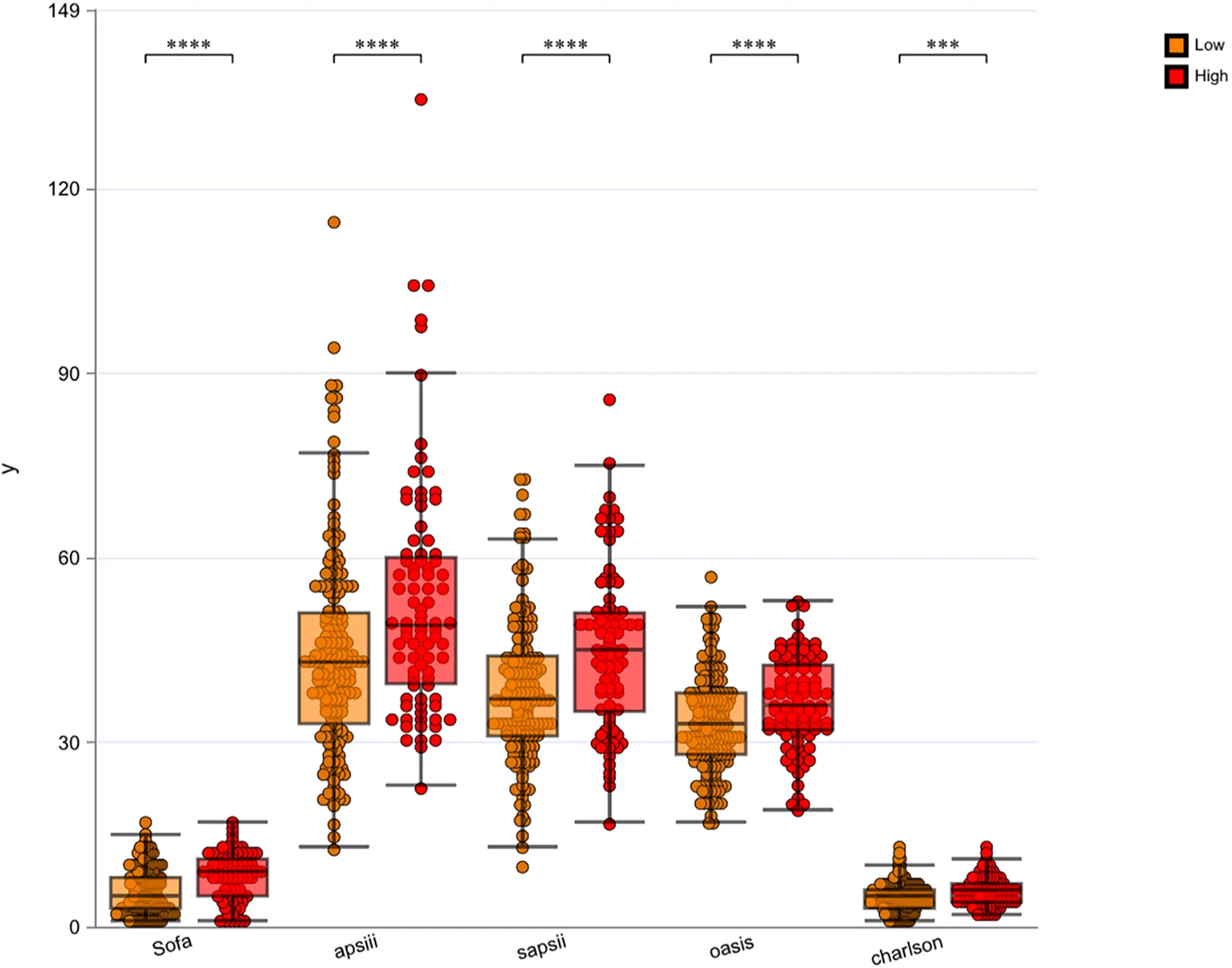
Relationship between RAR and Scoring Systems.

### 3.8 Kaplan–meier curves of RAR

Using the exploratory RAR cutoff value of 5.182, patients were divided into low- and high-RAR groups. In the exploratory Kaplan–Meier analysis, time zero was defined as ICU admission, and patients were followed until ICU death or ICU discharge. A significant difference in time to ICU death was observed between the two groups, with the high-RAR group showing lower ICU survival probability than the low-RAR group (unadjusted HR, 2.173; 95% CI, 1.045–4.520; *P* = 0.034) (Figure 10). Because ICU discharge alive may represent a competing event, these results should be interpreted as descriptive and exploratory, rather than definitive evidence of a causal or independent time-to-event association.

**Figure 10:**
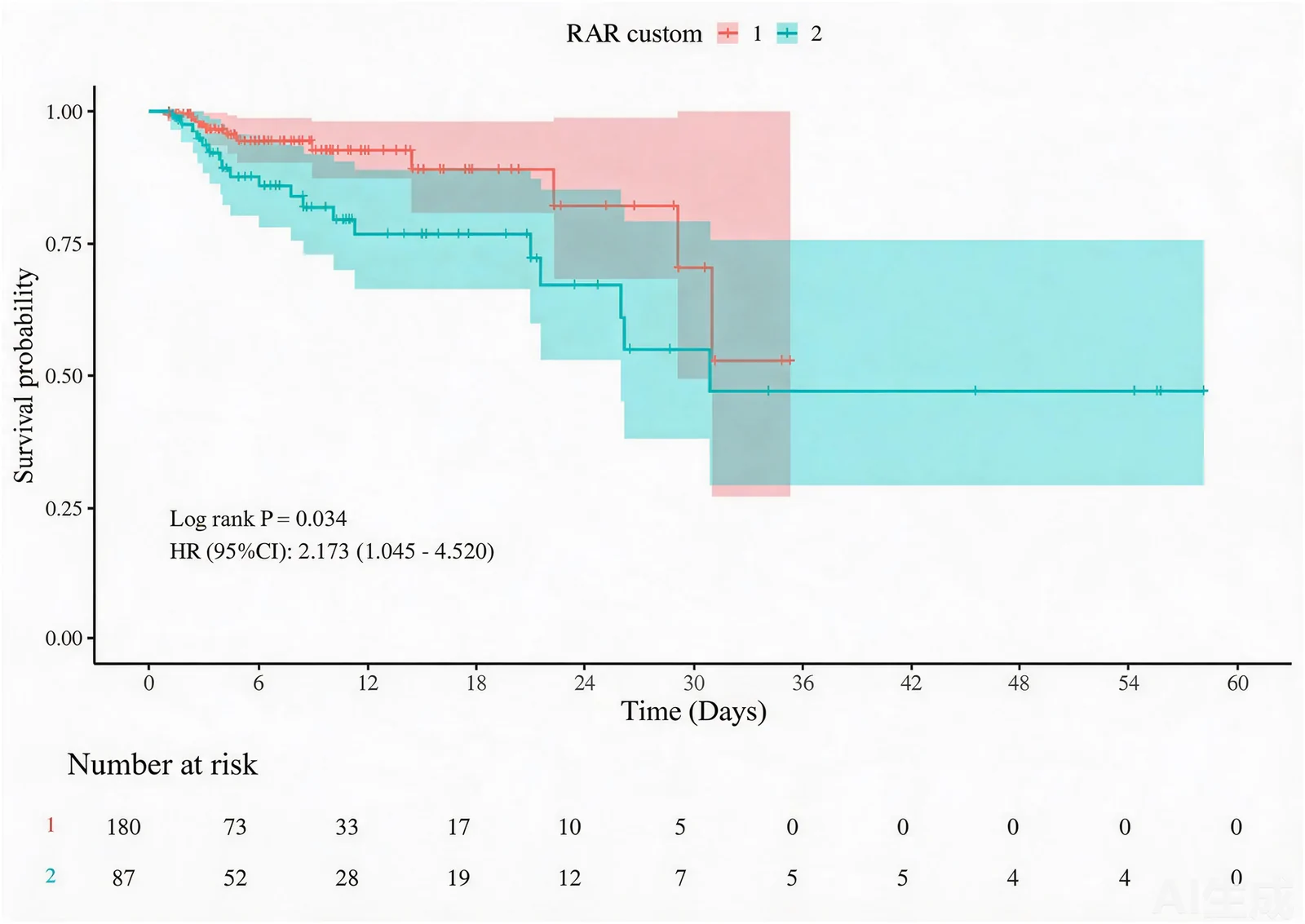
Kaplan-Meier curve for patients between the high and low RAR groups.

## 4 Discussion

In this study, we found that a higher RAR was associated with increased mortality risk among aortic dissection patients during ICU hospitalization. RAR may serve as a potential biomarker for predicting ICU mortality in this patient population.

RDW reflects variability in red blood cell size and is a potential prognostic marker in various diseases. Epidemiological studies show RDW predicts all-cause mortality and complications in the general population and specifically coronary artery disease (19). Serum albumin indicates nutritional status and inflammation and is linked to outcomes in coronary artery disease (20) and acute myocardial infarction (21). Even after adjusting for confounders, low albumin levels are associated with higher myocardial infarction risk (22). Albumin modulates inflammation through antioxidative and transport functions and is a recognized prognostic factor in critically ill patients (23, 24).

Recently, RAR has gained attention as a stable inflammatory marker for prognosis in critically ill patients. Studies have shown that higher RAR levels are associated with increased mortality in ICU sepsis patients (25), greater risk of acute kidney injury (26), and worse outcomes in burn patients independent of burn size (27). Elevated RAR is also linked to higher risks of end-stage renal disease, cardiovascular events, and mortality in chronic kidney disease (28). Additionally, RAR improves prediction of short-term mortality in acute pancreatitis (29) and independently predicts mortality in aneurysm patients (30).

Aortic dissection (AD) mainly affects people aged 55–80, with an incidence of about 40 per 100,000 and a recent trend toward younger patients, posing a public health challenge (31). Hypertension is the primary risk factor, especially if poorly controlled (32). Most patients are male (mean age ∼63) (11), but females have higher in-hospital mortality (33). Without prompt treatment within hours of symptom onset, mortality rises rapidly, with most deaths occurring within 30 days (34). Early identification of high-risk patients and timely intervention are therefore essential. This study aims to assess the clinical utility of RAR in predicting ICU outcomes in AD patients.

ROC curve analysis demonstrated that RAR has moderate predictive value for ICU mortality (AUC = 0.66, 95% CI: 0.55–0.76), with a sensitivity of 71.19% and specificity of 61.29%. However, the discriminatory performance of RAR should be interpreted cautiously. RAR showed only modest discrimination for ICU mortality in this study. Compared with its individual components, RAR numerically performed better than RDW alone but was similar to albumin alone, suggesting that the composite index may not provide a substantial advantage over albumin by itself. Moreover, adding RAR to the SOFA score resulted in only a small increase in AUC. The optimal cutoff value determined by the Youden index was 5.182, dividing patients into high- and low-RAR groups. Patients in the high-RAR group exhibited significantly lower hemoglobin, albumin, and red blood cell counts, alongside elevated levels of AST, creatinine, blood urea nitrogen, and INR. The predictive performance of RAR should be interpreted cautiously. Its discrimination for ICU mortality was modest, and the improvement in AUC after adding RAR to SOFA was small. In addition, although apparent calibration metrics for the SOFA + RAR model were acceptable, bootstrap internal validation was performed and optimism-corrected performance estimates are now reported, external validation is still required before broader clinical application. Furthermore, the cutoff value of 5.182 and its corresponding sensitivity and specificity were derived and assessed in the same relatively small cohort and should therefore be considered exploratory.

Inflammation and extracellular matrix degradation are considered key mechanisms in the formation of aortic dissection. False lumen formation and abnormal blood flow may cause organ hypoperfusion, cardiac tamponade, and aortic rupture (6), leading to metabolic disturbances, acidosis, and organ dysfunction, adversely affecting prognosis. The high RAR group had significantly longer ICU stays, and mechanical ventilation durations, as well as markedly higher SOFA, OASIS, SAPS II, APS III, and Charlson scores. RAR was positively correlated with these scoring systems, suggesting it may reflect disease severity. In the multivariable logistic regression analyses, the association between RAR and the primary outcome remained largely consistent across models with progressive adjustment for potential confounders. This suggests that the observed prognostic association of RAR was not fully explained by age, sex, underlying comorbidities, or selected inflammatory and organ function parameters. Nevertheless, the magnitude of the association was relatively modest, with the corresponding confidence intervals approaching the null value, and the findings should therefore be interpreted with caution. Moreover, given the single-center retrospective design, residual confounding and selection bias cannot be completely excluded. Further studies in larger, prospective, and multicenter cohorts are warranted to determine whether RAR provides independent and clinically meaningful prognostic information. Several mechanisms may explain the prognostic value of RAR in aortic dissection. First, elevated RDW reflects inflammatory activation, oxidative stress, and ineffective erythropoiesis, which are common in critically ill patients and may be exacerbated by acute vascular injury and perioperative stress (5, 6). Second, albumin is not only a marker of nutritional status but also a negative acute-phase protein with antioxidant and endothelial-protective effects (7, 23). Hypoalbuminemia may reflect systemic inflammation, capillary leakage, and poor physiological reserve, all of which are associated with adverse outcomes (20, 24). Therefore, a high RAR may capture the combined burden of inflammation, oxidative stress, malnutrition, and hematopoietic dysfunction, thereby identifying patients at higher risk of sepsis, AKI/RRT, and death. Our findings are consistent with the emerging literature suggesting that elevated RAR is associated with adverse outcomes across a range of cardiovascular and critical-care conditions. In addition to previously reported associations in sepsis and other critically ill populations, recent studies have demonstrated the clinical relevance of RAR in myocarditis and pericarditis (16, 17), while ICU cohort data have linked hematologic and metabolic indices including RAR to short-term mortality in sepsis patients (18). These data support the interpretation that RAR may represent a general marker of systemic inflammation, nutritional impairment, and illness severity rather than a biomarker specific to a single disease entity. This broader framework is in line with our findings in critically ill patients with acute aortic dissection.

Despite advances in the diagnosis and treatment of aortic dissection in recent years, mortality remains high. According to the IRAD database, the ICU mortality for TAAD patients is 22%, with a surgery-related mortality of 18% (35). This may reflect the severe and critical condition of patients upon admission, resulting in more complex treatment and prolonged disease courses. Elevated RAR may aid in early identification of patients requiring intensive monitoring or enhanced therapy. Kaplan–Meier survival curves showed that patients in the high RAR group had significantly lower survival rates than those in the low RAR group. RAR exhibited a linear relationship with postoperative sepsis and the need for RRT; both sepsis incidence and RRT requirement were significantly higher in the high RAR group. The RCS analyses further characterized the associations between RAR and the study outcomes. RAR showed an approximately linear association with both sepsis and use of RRT during ICU stay, with the risk increasing progressively across the observed range of RAR. In contrast, the association between RAR and AKI was nonlinear, suggesting that the relationship may vary across different levels of RAR. The different patterns observed across outcomes may reflect differences in the clinical and pathophysiological processes associated with sepsis, AKI, and use of RRT during ICU stay. However, these findings should be interpreted with caution. In particular, the nonlinear association between RAR and AKI does not establish a causal relationship and may be affected by residual confounding or the distribution of RAR within the study population. In this retrospective analysis of MIMIC-IV data, higher early-ICU RAR was associated with ICU mortality and other adverse ICU outcomes in patients with aortic dissection. However, discrimination was modest, improvement beyond SOFA was limited and not statistically significant, and external validation is required before any clinical application.

### 4.1 Limitations

This was a retrospective observational study, and causality cannot be inferred. Although the study used the large MIMIC-IV critical care database, the analysis was based on retrospectively extracted data rather than a dedicated prospective multicenter cohort. A major limitation is potential selection bias arising from the exclusion of patients without available RDW or albumin measurements. In the included-vs.-excluded comparison, included patients were more severely ill and had higher rates of sepsis, AKI, mechanical ventilation, and CRRT use, suggesting that the analytic cohort may not be representative of all ICU patients with aortic dissection in MIMIC-IV. Therefore, the observed associations should be interpreted cautiously and may not be generalizable to the broader ICU aortic dissection population. In addition, some clinically important variables, including detailed dissection classification, infection site, operative details, and certain ICU/hospitalization-related outcomes, were not fully available. The RAR cutoff value and its associated sensitivity and specificity were derived and assessed in the same relatively small cohort and should be considered exploratory. The survival analysis should also be interpreted cautiously because live ICU discharge may represent a competing event for ICU mortality rather than non-informative censoring. Finally, although bootstrap internal validation was performed, external validation was not conducted, and future multicenter prospective studies are warranted.

## 5 Conclusion

RAR demonstrates prognostic predictive ability in patients with aortic dissection. RAR was associated with ICU mortality and other adverse outcomes, with modest discriminatory ability for ICU mortality. Clinically, assessing RAR levels early upon ICU admission may facilitate early identification of high-risk patients to improve clinical outcomes.

## Data Availability

The datasets presented in this study can be found in online repositories. The names of the repository/repositories and accession number(s) can be found below: The data utilized in this study are derived from the Medical Information Mart for Intensive Care (MIMIC-IV) database, accessible via PhysioNet (https://doi.org/10.13026/hxp0-hg59).
