# Supplementary material for: Association of red cell distribution width-to-albumin ratio with ICU outcomes in patients with aortic dissection
Source: Front Cardiovasc Med. 2026 Oct 14;13:1909912. doi: 10.3389/fcvm.2026.1909912 (PMC13713700; doi:10.3389/fcvm.2026.1909912)
Supplement: Supplementary file 1 [file Table1.docx]

**Supplementary Table S1. Comparison of available baseline characteristics and outcomes between included patients and patients excluded due to missing RDW or albumin data**

| Variables | Total (n = 497) | Excluded groups (n = 230) | Included groups (n = 267) | Statistic | *P* |
| --- | --- | --- | --- | --- | --- |
|  |  |  |  |  |  |
| Age, M (Q₁, Q₃) | 69.00 (58.00, 79.00) | 69.00 (57.00, 79.00) | 70.00 (59.00, 79.00) | Z=-0.30 | 0.763 |
| White blood cell count (x10^9^/L), M (Q₁, Q₃) | 10.30 (8.00, 13.30) | 10.00 (8.10, 12.33) | 10.70 (7.90, 14.70) | Z=-1.65 | 0.100 |
| Red blood cell count (x10^12^/L), M (Q₁, Q₃) | 3.46 (2.90, 4.01) | 3.56 (3.08, 4.07) | 3.37 (2.79, 3.95) | Z=-2.22 | **0.026** |
| Platelet (x10^9^/L), M (Q₁, Q₃) | 154.00 (114.00, 207.00) | 165.00 (125.75, 218.25) | 145.00 (105.00, 197.00) | Z=-3.49 | **<.001** |
| Lactate (mmol/l), M (Q₁, Q₃) | 1.80 (1.20, 3.40) | 1.50 (1.10, 3.00) | 1.90 (1.30, 3.90) | Z=-3.04 | **0.002** |
| ALT, M (Q₁, Q₃) | 23.00 (15.00, 44.00) | 18.00 (13.00, 33.75) | 27.00 (16.00, 46.00) | Z=-2.78 | **0.005** |
| AST, M (Q₁, Q₃) | 37.00 (23.00, 78.75) | 26.00 (18.00, 39.00) | 41.00 (25.00, 85.00) | Z=-4.09 | **<.001** |
| BUN, M (Q₁, Q₃) | 18.00 (13.00, 23.00) | 16.00 (12.00, 21.00) | 19.00 (14.00, 24.00) | Z=-4.18 | **<.001** |
| Creatinine, M (Q₁, Q₃) | 1.00 (0.80, 1.30) | 0.90 (0.70, 1.20) | 1.10 (0.80, 1.40) | Z=-4.51 | **<.001** |
| SOFA, M (Q₁, Q₃) | 4.00 (2.00, 8.00) | 3.00 (1.00, 5.00) | 6.00 (3.00, 10.00) | Z=-8.20 | **<.001** |
| APSIII, M (Q₁, Q₃) | 39.00 (30.00, 50.00) | 34.00 (27.00, 43.00) | 44.00 (36.00, 55.00) | Z=-7.23 | **<.001** |
| SAPSII, M (Q₁, Q₃) | 36.00 (30.00, 44.00) | 32.00 (26.00, 39.00) | 39.00 (32.00, 48.00) | Z=-6.32 | **<.001** |
| OASIS, M (Q₁, Q₃) | 32.00 (26.00, 37.00) | 29.00 (24.00, 35.00) | 34.00 (29.00, 39.00) | Z=-6.19 | **<.001** |
| CHARLSON, M (Q₁, Q₃) | 5.00 (3.00, 6.00) | 4.00 (3.00, 6.00) | 5.00 (4.00, 7.00) | Z=-3.04 | **0.002** |
| Gender, n(%) |  |  |  | χ²=0.45 | 0.503 |
| F | 206 (41.45) | 99 (43.04) | 107 (40.07) |  |  |
| M | 291 (58.55) | 131 (56.96) | 160 (59.93) |  |  |
| Hypertension, n(%) |  |  |  | χ²=4.39 | **0.036** |
| 0 | 455 (91.55) | 211 (91.74) | 244 (91.39) |  |  |
| 1 | 315 (63.38) | 157 (68.26) | 158 (59.18) |  |  |
| Diabetes mellitus, n(%) |  |  |  | χ²=0.58 | 0.445 |
| 0 | 437 (87.93) | 205 (89.13) | 232 (86.89) |  |  |
| 1 | 60 (12.07) | 25 (10.87) | 35 (13.11) |  |  |
| HF, n(%) |  |  |  | χ²=11.71 | **<.001** |
| 0 | 407 (81.89) | 203 (88.26) | 204 (76.40) |  |  |
| 1 | 90 (18.11) | 27 (11.74) | 63 (23.60) |  |  |
| MI, n(%) |  |  |  | χ²=3.01 | 0.083 |
| 0 | 481 (96.78) | 226 (98.26) | 255 (95.51) |  |  |
| 1 | 16 (3.22) | 4 (1.74) | 12 (4.49) |  |  |
| MT, n(%) |  |  |  | χ²=0.28 | 0.599 |
| 0 | 430 (86.52) | 197 (85.65) | 233 (87.27) |  |  |
| 1 | 67 (13.48) | 33 (14.35) | 34 (12.73) |  |  |
| CKD, n(%) |  |  |  | χ²=3.97 | **0.046** |
| 0 | 412 (82.90) | 199 (86.52) | 213 (79.78) |  |  |
| 1 | 85 (17.10) | 31 (13.48) | 54 (20.22) |  |  |
| AF, n(%) |  |  |  | χ²=50.28 | **<.001** |
| 0 | 318 (63.98) | 185 (80.43) | 133 (49.81) |  |  |
| 1 | 179 (36.02) | 45 (19.57) | 134 (50.19) |  |  |
| Hepatitis, n(%) |  |  |  | χ²=0.03 | 0.867 |
| 0 | 492 (98.99) | 227 (98.70) | 265 (99.25) |  |  |
| 1 | 5 (1.01) | 3 (1.30) | 2 (0.75) |  |  |
| Pneumonia, n(%) |  |  |  | χ²=25.07 | **<.001** |
| 0 | 430 (86.52) | 218 (94.78) | 212 (79.40) |  |  |
| 1 | 67 (13.48) | 12 (5.22) | 55 (20.60) |  |  |
| Stroke, n(%) |  |  |  | χ²=0.02 | 0.888 |
| 0 | 455 (91.55) | 211 (91.74) | 244 (91.39) |  |  |
| 1 | 42 (8.45) | 19 (8.26) | 23 (8.61) |  |  |
| COPD, n(%) |  |  |  | χ²=0.01 | 0.922 |
| 0 | 448 (90.14) | 207 (90.00) | 241 (90.26) |  |  |
| 1 | 49 (9.86) | 23 (10.00) | 26 (9.74) |  |  |
| Ventilation, n(%) |  |  |  | χ²=12.73 | **<.001** |
| 0 | 66 (13.28) | 44 (19.13) | 22 (8.24) |  |  |
| 1 | 431 (86.72) | 186 (80.87) | 245 (91.76) |  |  |
| CRRT, n(%) |  |  |  | χ²=32.76 | **<.001** |
| 0 | 447 (89.94) | 226 (98.26) | 221 (82.77) |  |  |
| 1 | 50 (10.06) | 4 (1.74) | 46 (17.23) |  |  |
| Sepsis, n(%) |  |  |  | χ²=102.92 | **<.001** |
| 0 | 263 (52.92) | 178 (77.39) | 85 (31.84) |  |  |
| 1 | 234 (47.08) | 52 (22.61) | 182 (68.16) |  |  |
| AKI, n(%) |  |  |  | χ²=20.75 | **<.001** |
| 0 | 63 (12.68) | 46 (20.00) | 17 (6.37) |  |  |
| 1 | 434 (87.32) | 184 (80.00) | 250 (93.63) |  |  |
| ICU mortality, n(%) |  |  |  | χ²=3.62 | 0.057 |
| 0 | 444 (89.34) | 212 (92.17) | 232 (86.89) |  |  |
| 1 | 53 (10.66) | 18 (7.83) | 35 (13.11) |  |  |

Data are expressed as the median (25th–75th percentiles), or number (percentage). ALT, Alanine Aminotransferase; AST, Aspartate Aminotransferase; BUN, Blood Urea Nitrogen; HF, Heart failure; MI, Myocardial infarction; MT, Malignant tumor; CKD, Chronic kidney disease; AF, Atrial fibrillation; COPD, Chronic obstructive pulmonary disease; CRRT, continuous renal replacement therapy; AKI, acute kidney injury.
